# GENDER DIFFERENCES IN THE ASSOCIATIONS OF RECREATIONAL WALKING WITH INDOOR AND OUTDOOR FALLS AMONG OLDER ADULTS

**DOI:** 10.1093/geroni/igae098.2714

**Published:** 2024-12-31

**Authors:** Lingming Chen, Elizabeth Procter-Gray, Qun Le, Linda Churchill, Annabella Aguirre, Jie Cheng, Wenjun Li

**Affiliations:** University of Massachusetts Lowell, Lowell, Massachusetts, United States; University of Massachusetts Lowell, Lowell, Massachusetts, United States; University of Massachusetts Lowell, Lowell, Massachusetts, United States; University of Massachusetts Lowell, Lowell, Massachusetts, United States; University of Massachusetts Lowell, Lowell, Massachusetts, United States; University of Massachusetts Lowell, Lowell, Massachusetts, United States; University of Massachusetts Lowell, Lowell, Massachusetts, United States

## Abstract

Indoor and outdoor falls are different among older adults. Women and men may have different patterns of recreational walking and types of falls. This study examined the gender differences in the associations of recreational walking with risk for indoor and outdoor falls. This prospective cohort study enrolled 359 community-dwelling older adults aged 65-95 years old in central Massachusetts (2018-2020). Recreational walking at baseline was measured by the frequency of walking for exercise for at least 10 min in participants’ neighborhoods. Falls were reported on monthly fall calendars, and when a fall was reported, the circumstances were collected via telephone interview. Negative binomial models were used to estimate the gender differences in the associations of recreational walking with rates of indoor and outdoor falls, separately. Models were adjusting for sociodemographic variables, physical health, functional status, lifestyle behaviors, mental health, and the fear of falling. The results showed that less than half of participants did recreational walking at least once per week. Women had lower rates of indoor falls and outdoor falls than men (women vs. men: 34 vs. 51, 37 vs. 57 per 100 person-years). Women engaging in recreational walking at least once per week had 86% lower indoor fall rates (IRR (95% CI): 0.14 (0.05, 0.44)) than those who did not. No significant associations were found between recreational walking and outdoor falls. In conclusion, for older women, but not men, higher frequency of recreational walking was associated with lower rates of indoor falls.

